# Repurposing therapy of ibrexafungerp vulvovaginal candidiasis drugs as cancer therapeutics

**DOI:** 10.3389/fphar.2024.1428755

**Published:** 2024-06-27

**Authors:** Tedi Rustandi, Abdul Mahmud Yumassik, Fitrah Shafran Ilahi, Riza Alfian, Erna Prihandiwati, Yugo Susanto, Yudi Hardi Susilo, Maria Ulfah, Faizatun Faizatun

**Affiliations:** ^1^ Pharmacy, Sekolah Tinggi Ilmu Kesehatan ISFI Banjarmasin, Banjarmasin, Indonesia; ^2^ Department of Pharmaceutical Technology, Faculty of Pharmacy, Pancasila University, Jakarta, Indonesia

**Keywords:** antifungal, anticancer, computational screening, glucan synthase inhibitor, ROS, triterpenoid, UBE2M, autophagy

## 1 Introduction

The prevalence of cancer in 2022, according to World Health Organization (WHO) data, is 20 million new cases and 9.7 deaths. The comparison of death rates based on gender is that 1 in 9 men and 2 in 12 women die from cancer (WHO, 2024). New cancer cases in the United States (US) in 2024 will be 2,001,140, with 611,720 resulting in death (Siegel et al., 2024).

Cancer is one of the leading causes of death worldwide, with the rate of adoption of new drugs likely to be slower in clinical practice than expected. New drug development takes a long time, with an average of 13 years at a cost of ∼USD 2–3 billion (Zhang et al., 2020). This condition has global health and financial burdens (Roth et al., 2018). Discovery and development of new drugs to overcome this need to be done.

The drug repurposing method is a promising approach that will accelerate the research and development cycle. This approach is more effective in terms of cost and time than drug research and development using the *de novo* drug discovery approach (Tran and Prasad, 2020). Ibrexafungerp, approved by the FDA in 2021 as an antifungal derived from natural-product-based small compounds, has excellent potential to be developed using repurposing techniques to become a drug with other functions (Xu et al., 2022). The success of repurposing techniques in the development of anticancer drugs that have been approved by the FDA, such as a combination of aspirin, the antibiotic doxycycline, mifepristone, and the amino acid lysine, is used to prevent cancer metastasis (Wan et al., 2015).

The method used in this opinion article is a literature review. The literature review process uses Pubmed, Scopus, and Springer databases with criteria for articles published from 2015–2024. The article search method uses the query “repurposing therapy” AND/OR “ibrexafungerp” AND/OR “vulvovaginal candidiasis” AND/OR “cancer” AND/OR “computational screening” AND/OR “glucan synthase inhibitor” AND/OR “triterpenoid” AND/OR “ROS” AND/OR “siRNA” AND/OR “cancer mechanism” AND/OR “Tools” AND/OR “Computational” AND/OR “Artificial Intelligence” AND/OR “In Silico” AND/OR “Deep Learning” AND/OR “Machine Learning” AND/OR “bioinformatics.”

## 2 Mechanism ibrexafungerp for anticancer

Ibrexafungerp has antifungal activity by inhibiting (1,3)-β-D-glucan synthase (Apgar et al., 2021). This mechanism gives Ibrexafungerp a good toxicity profile in host cells. The pharmacokinetic profile of Ibrexafungerp is well-classified, with the ability to penetrate tissues and organs, such as the liver, lungs, and skin. This pharmacokinetic profile is influenced by the structure of Ibrexafungerp, which has a core phenanthropyran carboxylic acid ring system at position 15 and 2-amino-2,3,3-trimethyl-butyl ether at position 14, both of which are derivatives of the naturally occurring hemiacetal triterpene glycoside enfumafungin. The pharmacokinetic profile in animals shows that Ibrexafungerp has a 30%–50% bioavailability when administered orally and has poor penetration into the central nervous system. *In vitro* studies show hydroxylation metabolism by the CYP3A4 isoenzyme with primary excretion via bile. The steady-state volume of distribution (Vss) profile in humans averages 600 L with high binding to protein, mainly albumin (Apgar et al., 2021; Angulo et al., 2022).

The potential of Ibrexafungerp as a cancer therapeutics is based on the use of antifungals, which have been used as anticancer agents. Antifungals with anticancer activity include itraconazole, rapamycin, griseofulvin, clotrimazole, ciclopirox, and nannocystin A (Li et al., 2022; Mohi-ud-din et al., 2023). The mechanisms of antifungal drugs that act as anticancers include the function of increasing autophagy, reducing angiogenesis, increasing tumor regression, and reducing metastasis (Mohi-ud-din et al., 2023).

Ibrexafungerp has a mechanism as a non-competitive glucan synthase inhibitor and the exact mechanism as echinocandins as an antifungal (Jallow and Govender, 2021; Shi et al., 2023; Kumar et al., 2024). Ibrexafungerp’s activity includes a broad-spectrum anti-candida fungicide against species resistant to azole drugs. Capable *Candida* species associated with ibrexafungerp activity include auris, dubliniensis, glabrata, guilliermondii, keyfr, krusei, lusitaniae, parapsilosis, and tropicalis (Phillips et al., 2023). Activity as a broad-spectrum antifungal, such as *Candida* species, indicates that ibrexafungerp may have anticancer activity. The anticancer activity of broad-spectrum antifungals such as the triazole group, namely, itraconazole, is related to the mechanism of molecular smoothened (SMO) D477G mutations, sterol carrier protein 2 (SCP2), voltage-dependent anion channel 1 (VDAC1), and Niemann-Pick Type C 1 (NPC1) (Weng et al., 2023).

**FIGURE 1:**
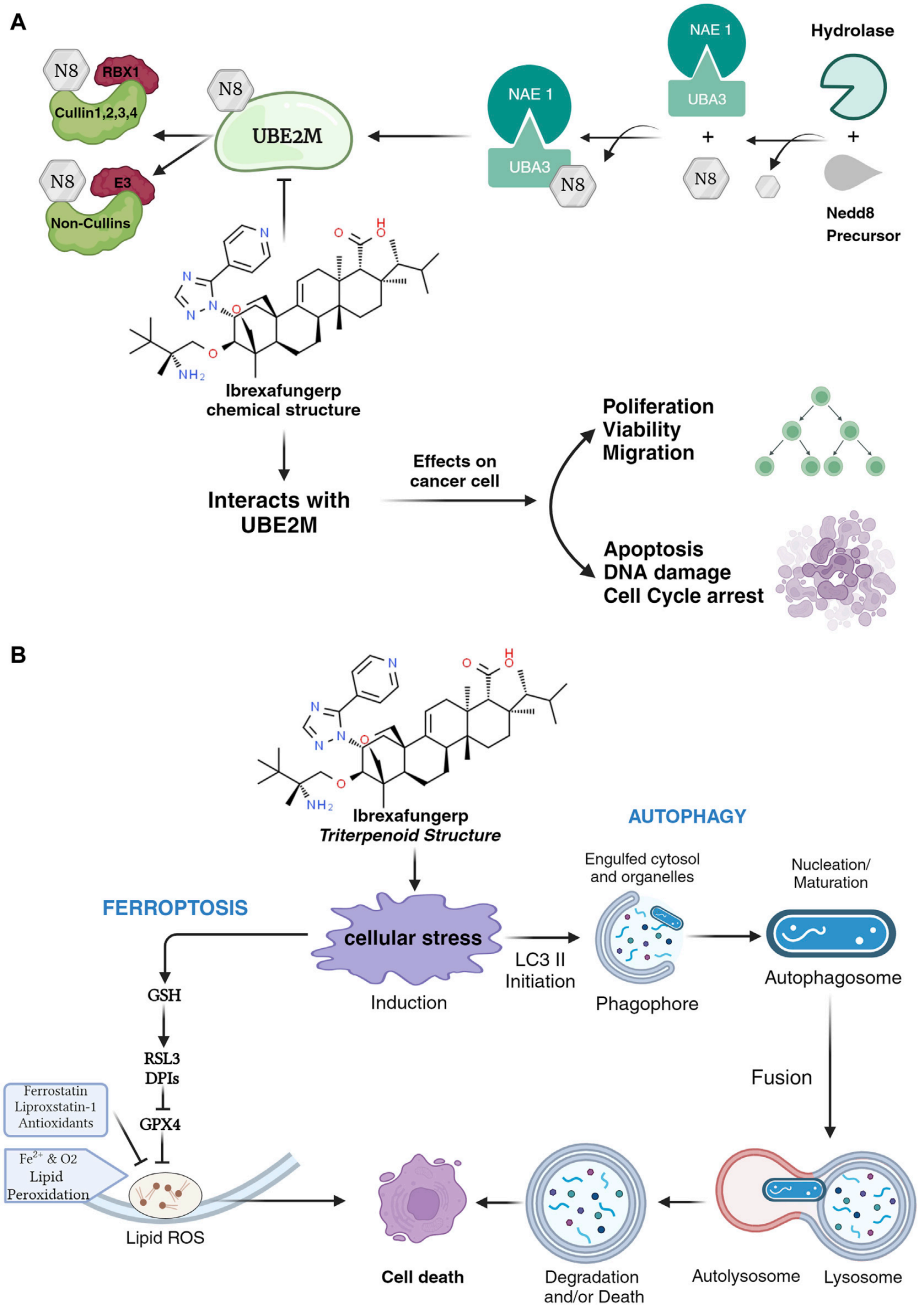
**(A)** Mechanism of Ibrexafungerp in inhibiting the neddylation process and **(B)** Mechanism of Ibrexafungerp as a ROS modulator.

The mechanism of ibrexafungerp has the same action as micafungin, which is one of the echinocandin classes of antifungal agents. The mechanisms of action of Ibrexafungerp and micafungin as antifungals may have mechanisms similar to anticancer. The predicted mechanism of ibrexafungerp is to inhibit the neddylation process by stabilizing ubiquitin-conjugating enzyme 2 M (UBE2M). This enzyme is essential in molecular mechanisms such as DNA damage, apoptosis, and cell proliferation (Mamun et al., 2023a). The prediction of the Ibrexafungerp mechanism can be seen in Figure 1A (Mamun et al., 2023b; Mamun et al., 2023a; Yu et al., 2020b; Zheng et al., 2021; Zhou et al., 2023).

Prediction of the mechanism of ibrexafungerp as a UBE2M inhibitor can inhibit the neddylation pathway which can reduce tumor-promoting factors and increase levels of tumor suppressors thereby improving the occurrence of tumors and prognosis (Zheng et al., 2021). Anticancers that target UBE2M in the neddylation process play a role in posttranslational modification mechanisms and target protein activity. The neddylation process begins with NEDD8 which is activated by E1 NEDD8-activating enzyme (NAE-consists of NAE1 and UBA3). This activation process results in the formation of the thioester-linked E1-NEDD8 complex which is then transferred to the NEDD8-conjugating enzyme (E2)/UBE2M (Yu et al., 2020b; Zheng et al., 2021). Ibrexafungerp inhibits the NEDD8 mechanism in UBE2M so that it cannot proceed to the next stage, namely, transferring NEDD8 from charged E2 to lysine residues in its target (Zhou et al., 2023).

Ibrexafungerp has a structure that belongs to the triterpenoid class (Angulo et al., 2022; Kumar et al., 2024). The triterpenoid group has the potential to be a cancer chemotherapy agent with a mechanism as a reactive oxygen species (ROS) modulator that can regulate cell survival and function. The impact of ROS on cancer cells is the mechanism of autophagy and ferroptosis (Endale et al., 2023; Jiang et al., 2021; Lee et al., 2023; Ling et al., 2022; Zeng et al., 2023). Autophagy works by causing cellular lipid accumulation and, ultimately, cell death. Another mechanism is inducing ferroptosis, which can cause increased chemosensitivity to chemotherapy drugs that are used to treat cancer cells. The mechanism of ibrexafungerp as a ROS modulator can be seen in Figure 1B (Ling et al., 2022).

## 3 Computational approaches ibrexafungerp

The development of ibrexafungerp as a cancer therapeutic can be done through 2 methods: experimental screening and computational (virtual) screening (Oliveira et al., 2023; Prada Gori et al., 2023; Weth et al., 2024). Experimental screening involves *in vivo* and *in vitro* research with drug-based phenotypic screens and target-based high throughput assays. Computational (virtual) screening methods include signature matching (-omics data), artificial intelligence (machine learning and deep learning), GWAS disease/target associations, and chemical similarity and molecular docking (Weth et al., 2024). A virtual screening server that can be used in computational approaches in the development of drug repurposing research, namely, DrugRep. The use of DrugRep in drug repurposing research uses receptor-based and ligand-based screening systems (Gan et al., 2023). Several tools can be used to develop anticancer from Ibrexafungerp, some of which can be seen in Table 1.

**TABLE 1:** Computational tools in cancer research.

Tools	Function	Web link	Reference
The cancer proteome Atlas V3.0 (TCPA V3.0)	Supports research to visualize and analyze Reverse phase protein arrays (RPPA) data	http://tcpaportal.org	Chen et al. (2019)
DemixTallmaterial	Supports research to estimate the proportion of specific cell types (tumor, stromal and immune cells) simultaneously	https://github.com/wwylab/DeMixTallmaterials	Wang et al. (2018)
The Cancer Genom Atlas (TCGA)	Supports research as a Platform that has a catalog of analysis data in large groups to study cancer genetics	https://www.genome.gov/Funded-Programs-Projects/Cancer-Genome-Atlas	Tomczak et al. (2015)
Tumor MAP	Supports research for visualization and interactive analysis in exploring patterns between tumor cells arranged relative to each other based on their molecules	https://tumormap.ucsc.edu/	Gabriel et al. (2020)
SurvNet	Using one of the artificial intelligence (AI) methods, namely, Deep Neural Network (DNN), which is used to analyze lung cancer survival	https://bioinformatics.mdanderson.org/SurvNet/	Wang et al. (2021)
METABRIC, HapMap, Lincs, KEGG, DrugBank	Identification of drug repurposing results of the best analysis for each breast cancer subtype	Not Available (NA)	Firoozbakht et al. (2022)
shinyDeepDR	The study of personalization of cancer treatment through analysis of drug response to differences in genomic attributes	https://shiny.crc.pitt.edu/shinydeepdr/	Wang et al. (2024)
iODA	Tools used for heterogeneous multi-omics data analysis	http://www.sysbio.org.cn/iODA	Yu et al. (2020a)
MLSP	Bioinformatic analysis tools in breast cancer research to predict molecular subtypes and prognosis	https://sujiezhulab.shinyapps.io/BRCA/	Zhu et al. (2022)
DEBay	A tool that can be used in cancer research related to gene expression through quantitative PCR data deconvolution	https://sourceforge.net/projects/debay	Devaraj and Bose (2020)
The HPV Induced Cancer Resource (THInCR)	Tools used to explore the impact of HPV on cellular gene expression (mRNA and microRNA), changes in gene methylation, and their relationship to patient survival and features of the immune landscape	https://thincr.ca/	Salnikov et al. (2022)
DAX-Net	The model that utilizes Convolutional Neural Network (CNN) and Transformer network for multiclass cancer classification	https://github.com/QuIIL/DAX-Net	Bui et al. (2024)
ExplORRNet	Tool for research related to miRNA expression profiles	https://mirna.cs.ut.ee	Lawarde et al. (2024)

## 4 Conclusion

Ibrexafungerp is predicted to have two anticancer mechanisms. The anticancer mechanism is obtained by inhibiting the neddylation stage by stabilizing UBE2M, and Ibrexafungerp acts as a ROS modulator, which acts through cell death mechanisms with autophagy and ferroptosis.

## References

[B1] Angulo D. A. Alexander B. Rautemaa-Richardson R. Alastruey-Izquierdo A. Hoenigl M. Ibrahim A. S. (2022). Ibrexafungerp, a novel triterpenoid antifungal in development for the treatment of mold infections. J. Fungi 8 (11), 1121. 10.3390/jof8111121 PMC9695964 36354888

[B2] Apgar J. M. Wilkening R. R. Parker D. L. Meng D. Wildonger K. J. Sperbeck D. (2021). Ibrexafungerp: an orally active β-1,3-glucan synthesis inhibitor. Bioorg. Med. Chem. Lett. 32, 127661. 10.1016/j.bmcl.2020.127661 33160023

[B3] Bui D. C. Song B. Kim K. Kwak J. T. (2024). DAX-Net: a dual-branch dual-task adaptive cross-weight feature fusion network for robust multi-class cancer classification in pathology images. Comput. Methods Programs Biomed. 248, 108112. 10.1016/j.cmpb.2024.108112 38479146

[B4] Chen M. J. M. Li J. Wang Y. Akbani R. Lu Y. Mills G. B. (2019). TCPA v3.0: an integrative platform to explore the pan-cancer analysis of functional proteomic data. Mol. Cell. Proteomics 18 (8), S15–S25. 10.1074/mcp.RA118.001260 31201206 PMC6692772

[B5] Devaraj V. Bose B. (2020). DEBay: a computational tool for deconvolution of quantitative PCR data for estimation of cell type-specific gene expression in a mixed population. Heliyon 6 (7), e04489. 10.1016/j.heliyon.2020.e04489 32728643 PMC7381708

[B6] Endale H. T. Tesfaye W. Mengstie T. A. (2023). ROS induced lipid peroxidation and their role in ferroptosis. Front. Cell Dev. Biol. 11, 1226044. 10.3389/fcell.2023.1226044 37601095 PMC10434548

[B7] Firoozbakht F. Rezaeian I. Rueda L. Ngom A. (2022). Computationally repurposing drugs for breast cancer subtypes using a network-based approach. BMC Bioinforma. 23 (1), 143. 10.1186/s12859-022-04662-6 PMC9020161 35443626

[B8] Gabriel A. A. G. Mathian E. Mangiante L. Voegele C. Cahais V. Ghantous A. (2020). A molecular map of lung neuroendocrine neoplasms. GigaScience 9 (11), giaa112. 10.1093/gigascience/giaa112 33124659 PMC7596803

[B9] Gan J. H. Liu J. X. Liu Y. Chen S. W. Dai W. T. Xiao Z. X. (2023). DrugRep: an automatic virtual screening server for drug repurposing. Acta Pharmacol. Sin. 44 (4), 888–896. 10.1038/s41401-022-00996-2 36216900 PMC9549438

[B10] Jallow S. Govender N. P. (2021). Ibrexafungerp: a first-in-class oral triterpenoid glucan synthase inhibitor. J. Fungi 7 (3), 163. 10.3390/jof7030163 PMC7996284 33668824

[B11] Jiang M. Hu R. Yu R. Tang Y. Li J. (2021). A narrative review of mechanisms of ferroptosis in cancer: new challenges and opportunities. Ann. Transl. Med. 9 (20), 1599. 10.21037/atm-21-4863 34790805 PMC8576726

[B12] Kumar V. Huang J. Dong Y. Hao G. F. (2024). Targeting Fks1 proteins for novel antifungal drug discovery. Trends Pharmacol. Sci. 45 (4), 366–384. 10.1016/j.tips.2024.02.007 38493014

[B13] Lawarde A. Sharif Rahmani E. Nath A. Lavogina D. Jaal J. Salumets A. (2024). ExplORRNet: an interactive web tool to explore stage-wise miRNA expression profiles and their interactions with mRNA and lncRNA in human breast and gynecological cancers. Non-Coding RNA Res. 9 (1), 125–140. 10.1016/j.ncrna.2023.10.006 PMC10686811 38035042

[B14] Lee S. Hwang N. Seok B. G. Lee S. Lee S. J. Chung S. W. (2023). Autophagy mediates an amplification loop during ferroptosis. Cell Death Dis. 14 (7), 464. 10.1038/s41419-023-05978-8 37491375 PMC10368698

[B15] Li C. L. Fang Z. X. Wu Z. Hou Y. Y. Wu H. T. Liu J. (2022). Repurposed itraconazole for use in the treatment of malignancies as a promising therapeutic strategy. Biomed. Pharmacother. 154, 113616. 10.1016/j.biopha.2022.113616 36055112

[B16] Ling T. Boyd L. Rivas F. (2022). Triterpenoids as reactive oxygen species modulators of cell fate. Chem. Res. Toxicol. 35 (4), 569–584. 10.1021/acs.chemrestox.1c00428 35312315 PMC9019815

[B17] Mamun M. Liu Y. Geng Y. P. Zheng Y. C. Gao Y. Sun J. G. (2023). Discovery of neddylation E2s inhibitors with therapeutic activity. Oncogenesis 12 (1), 45. 10.1038/s41389-023-00490-2 37717015 PMC10505188

[B18] Mamun M. A. A. Liu S. Zhao L. Zhao L. Li Z.-R. Shen D. (2023). Micafungin: a promising inhibitor of UBE2M in cancer cell growth suppression. Eur. J. Med. Chem. 260, 115732. 10.1016/j.ejmech.2023.115732 37651876

[B19] Mohi-ud-din R. Chawla A. Sharma P. Mir P. A. Potoo F. H. Reiner Ž. (2023). Repurposing approved non-oncology drugs for cancer therapy: a comprehensive review of mechanisms, efficacy, and clinical prospects. Eur. J. Med. Res. 28 (1), 345. 10.1186/s40001-023-01275-4 37710280 PMC10500791

[B20] Oliveira T. Silva M. Maia E. Silva A. Taranto A. (2023). Virtual screening algorithms in drug discovery: a review focused on machine and deep learning methods. Drugs Drug Candidates 2 (2), 311–334. 10.3390/ddc2020017

[B21] Phillips N. A. Rocktashel M. Merjanian L. (2023). Ibrexafungerp for the treatment of vulvovaginal Candidiasis: design, development and place in therapy. Drug Des. Dev. Ther. 17, 363–367. 10.2147/DDDT.S339349 PMC9921437 36785761

[B22] Prada Gori D. N. Ruatta S. Fló M. Alberca L. N. Bellera C. L. Park S. (2023). Drug repurposing screening validated by experimental assays identifies two clinical drugs targeting SARS-CoV-2 main protease. Front. Drug Discov. 2. 10.3389/fddsv.2022.1082065

[B23] Roth G. A. Abate D. Abate K. H. Abay S. M. Abbafati C. Abbasi N. (2018). Global, regional, and national age-sex-specific mortality for 282 causes of death in 195 countries and territories, 1980–2017: a systematic analysis for the Global Burden of Disease Study 2017. Lancet 392 (10159), 1736–1788. 10.1016/S0140-6736(18)32203-7 30496103 PMC6227606

[B24] Salnikov M. Gameiro S. F. Zeng P. Y. F. Barrett J. W. Nichols A. C. Mymryk J. S. (2022). The HPV Induced Cancer Resource (THInCR): a suite of tools for investigating HPV-Dependent human carcinogenesis. MSphere 7 (4), e0031722. 10.1128/msphere.00317-22 35950764 PMC9429961

[B25] Shi Z. Zhang J. Tian L. Xin L. Liang C. Ren X. (2023). A comprehensive overview of the antibiotics approved in the last two decades: retrospects and prospects. Molecules 28 (4), 1762. 10.3390/molecules28041762 36838752 PMC9962477

[B26] Siegel R. L. Giaquinto A. N. Jemal A. (2024). Cancer statistics, 2024. CA A Cancer J. Clin. 74 (1), 12–49. 10.3322/caac.21820 38230766

[B27] Tomczak K. Czerwińska P. Wiznerowicz M. (2015). The Cancer Genome Atlas (TCGA): an immeasurable source of knowledge. Wspolczesna Onkol. 1A, A68–A77. 10.5114/wo.2014.47136 PMC4322527 25691825

[B28] Tran A. A. Prasad V. (2020). Drug repurposing for cancer treatments: a well-intentioned, but misguided strategy. Lancet Oncol. 21 (9), 1134–1136. 10.1016/S1470-2045(20)30424-1 32888447

[B29] Wan L. Dong H. Xu H. Ma J. Zhu Y. Lu Y. (2015). Aspirin, lysine, mifepristone and doxycycline combined can effectively and safely prevent and treat cancer metastasis: prevent seeds from gemmating on soil. Oncotarget 6 (34), 35157–35172. 10.18632/oncotarget.6038 26459390 PMC4742096

[B30] Wang J. Chen N. Guo J. Xu X. Liu L. Yi Z. (2021). SurvNet: a novel deep neural network for lung cancer survival analysis with missing values. Front. Oncol. 10, 588990. 10.3389/fonc.2020.588990 33552965 PMC7855857

[B31] Wang L. J. Ning M. Nayak T. Kasper M. J. Monga S. P. Huang Y. (2024). shinyDeepDR: a user-friendly R Shiny app for predicting anti-cancer drug response using deep learning. Patterns 5 (2), 100894. 10.1016/j.patter.2023.100894 38370127 PMC10873157

[B32] Wang Z. Cao S. Morris J. S. Ahn J. Liu R. Tyekucheva S. (2018). Transcriptome deconvolution of heterogeneous tumor samples with immune infiltration. IScience 9, 451–460. 10.1016/j.isci.2018.10.028 30469014 PMC6249353

[B33] Weng N. Zhang Z. Tan Y. Zhang X. Wei X. Zhu Q. (2023). Repurposing antifungal drugs for cancer therapy. J. Adv. Res. 48, 259–273. 10.1016/j.jare.2022.08.018 36067975 PMC10248799

[B34] Weth F. R. Hoggarth G. B. Weth A. F. Paterson E. White M. P. J. Tan S. T. (2024). Unlocking hidden potential: advancements, approaches, and obstacles in repurposing drugs for cancer therapy. Br. J. Cancer 130 (5), 703–715. 10.1038/s41416-023-02502-9 38012383 PMC10912636

[B35] WHO (2024) Global cancer burden growing, amidst mounting need for services. Lyon, France, Geneva, Switzerland: World Health Organization. Available at: https://www.who.int/news/item/01-02-2024-global-cancer-burden-growing--amidst-mounting-need-for-services.

[B36] Xu Z. Eichler B. Klausner E. A. Duffy-Matzner J. Zheng W. (2022). Lead/Drug discovery from natural resources. Molecules 27 (23), 8280. 10.3390/molecules27238280 36500375 PMC9736696

[B37] Yu C. Qi X. Lin Y. Li Y. Shen B. (2020). iODA: an integrated tool for analysis of cancer pathway consistency from heterogeneous multi-omics data. J. Biomed. Inf. 112, 103605. 10.1016/j.jbi.2020.103605 33096244

[B38] Yu Q. Jiang Y. Sun Y. (2020). Anticancer drug discovery by targeting cullin neddylation. Acta Pharm. Sin. B 10 (5), 746–765. 10.1016/j.apsb.2019.09.005 32528826 PMC7276695

[B39] Zeng X. Y. Qiu X. Z. Liang S. M. Huang J. A. Liu S. Q. Wu J. N. (2023). Interaction mechanisms between autophagy and ferroptosis: potential role in colorectal cancer. World J. Gastrointest. Oncol. 15 (7), 1135–1148. 10.4251/wjgo.v15.i7.1135 37546557 PMC10401467

[B40] Zhang Z. Zhou L. Xie N. Nice E. C. Zhang T. Cui Y. (2020). Overcoming cancer therapeutic bottleneck by drug repurposing. Signal Transduct. Target. Ther. 5 (1), 113. 10.1038/s41392-020-00213-8 32616710 PMC7331117

[B41] Zheng Y. C. Guo Y. J. Wang B. Wang C. Mamun M. A. A. Gao Y. (2021). Targeting neddylation E2s: a novel therapeutic strategy in cancer. J. Hematol. Oncol. 14 (1), 57. 10.1186/s13045-021-01070-w 33827629 PMC8028724

[B42] Zhou L. Lin X. Zhu J. Zhang L. Chen S. Yang H. (2023). NEDD8-conjugating enzyme E2s: critical targets for cancer therapy. Cell Death Discov. 9 (1), 23. 10.1038/s41420-023-01337-w 36690633 PMC9871045

[B43] Zhu J. Kong W. Huang L. Wang S. Bi S. Wang Y. (2022). MLSP: a bioinformatics tool for predicting molecular subtypes and prognosis in patients with breast cancer. Comput. Struct. Biotechnol. J. 20, 6412–6426. 10.1016/j.csbj.2022.11.017 36467575 PMC9685393

